# Supplementary material for: Chitosan/Silver Nanoparticle/Graphene Oxide Nanocomposites with Multi-Drug Release, Antimicrobial, and Photothermal Conversion Functions
Source: Materials (Basel). 2021 Apr 30;14(9):2351. doi: 10.3390/ma14092351 (PMC8124926; doi:10.3390/ma14092351)
Supplement: Supplementary file 1 [file materials-14-02351-s001.zip › materials-1193360-supplementary.pdf]

materials

Article

# Chitosan/Silver Nanoparticle/Graphene Oxide Nanocomposites with Multi-Drug Release, Antimicrobial, and Photothermal Conversion Functions

**Zheng Su [1,2], Daye Sun [2], Li Zhang [3], Miaomiao He [3], Yulin Jiang [3], Bronagh Millar [2], Paula Douglas [2], Davide Mariotti [4], Paul Maguire [4] and Dan Sun [2,*]**

[1] Department of orthopedics, The First Affiliated Hospital of USTC, Division of Life Sciences and Medicine, University of Science and Technology of China, Hefei 230001, China; suz924@mail.ustc.edu.cn
[2] School of Mechanical & Aerospace Engineering, Queens University Belfast, Belfast BT9 5AH, UK; dsun03@qub.ac.uk (D.S.); b.millar@qub.ac.uk (B.M.); p.douglas@qub.ac.uk (P.D.)
[3] Research Center for Nano-Biomaterials, Analytical & Testing Center, Sichuan University, Chengdu 610065, China; zhangli9111@126.com (L.Z.); hemmiao@126.com (M.H.); yljiangss@126.com (Y.J.);
[4] Nanotechnology and Integrated Bioengineering Center (NIBEC), Ulster University, Co Antrim BT37 OQB, UK; d.mariotti@ulster.ac.uk (D.M.); pd.maguire@ulster.ac.uk (P.M.)
* Correspondence: d.sun@qub.ac.uk; Tel.: +44-(0)-28-909-74-701

**Table S1.** Effect of AgNPs and GO addition on the thermal properties of pure CS, CS/AgNPs and CS/AgNPs/GO scaffold.

| | Endothermic peak | | | Exothermic peak | | | |
|---|---|---|---|---|---|---|---|
| | $T_{endo}$(°C) | ΔH(J/g) | $S_{endo}$(mJ) | $T_{exo}$(°C) | ΔH(J/g) | $S_{exo}$(mJ) | $T_g$(°C) |
| Pure CS | 150.69 | 52.92 | 169.34 | 297.07 | -105.16 | -336.51 | 134.70 |
| CS/AgNPs | 152.85 | 55.04 | 176.14 | 291.25 | -103.69 | -331.81 | 138.89 |
| CS/RB-AgNPs/GO-0.5 | 154.01 | 54.79 | 169.86 | 297.58 | -119.71 | -371.09 | 140.39 |
| CS/RB-AgNPs/GO-1.5 | 150.85 | 52.73 | 174.00 | 300.57 | -101.25 | -334.12 | 141.91 |

Note: ΔH(J/g) = heat of fusion

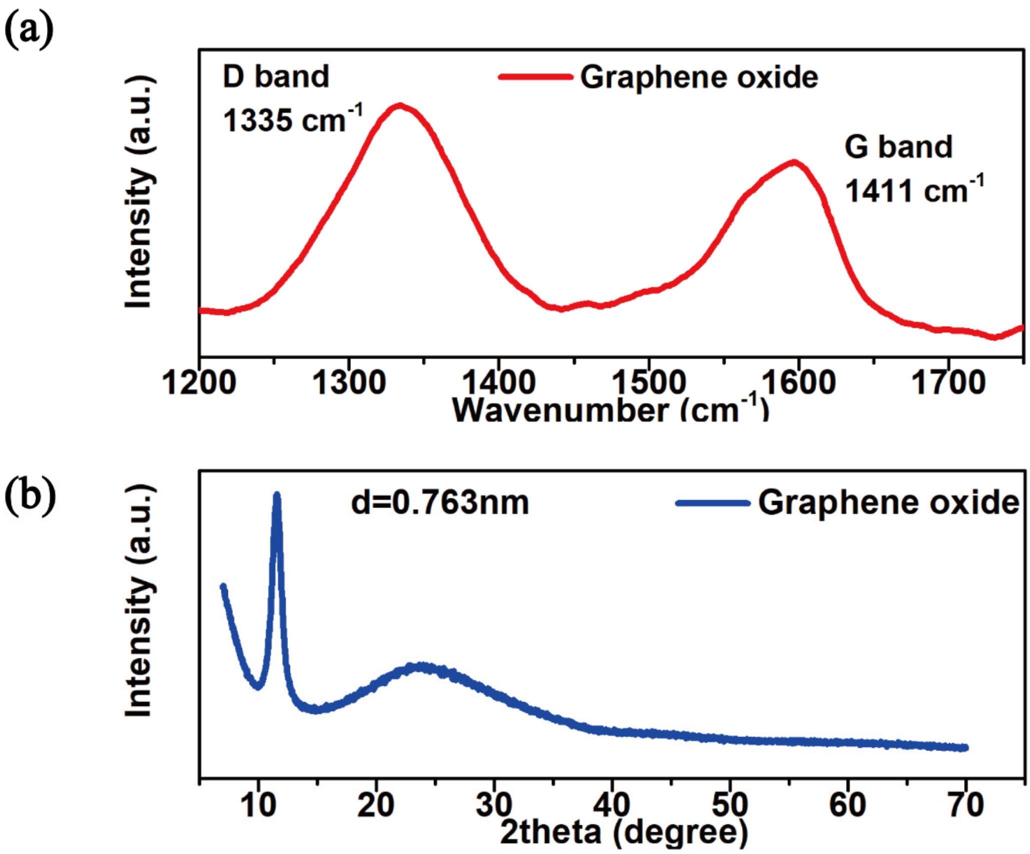


**Figure S1.** (a) The Raman spectra, and (b) X-ray diffraction (XRD) pattern of GO.

The typical D band (1335 $cm^{-1}$) and G band (1411 $cm^{-1}$) of GO are shown in Fig. S1 (a), representing the aromatic structure and edge of GO and the vibration of the $sp^2$ carbon atoms, respectively [1, 2]. In Fig. S1 (b), the GO presented a peak at 11.59 degree (002) with d-spacing of 0.763 nm, which matches the well-known XRD pattern of graphene oxide [3]. Because of the short-range order in the stacked graphene like sheets, the GO also shows a weak broad peak at around 24.31 degrees [4].

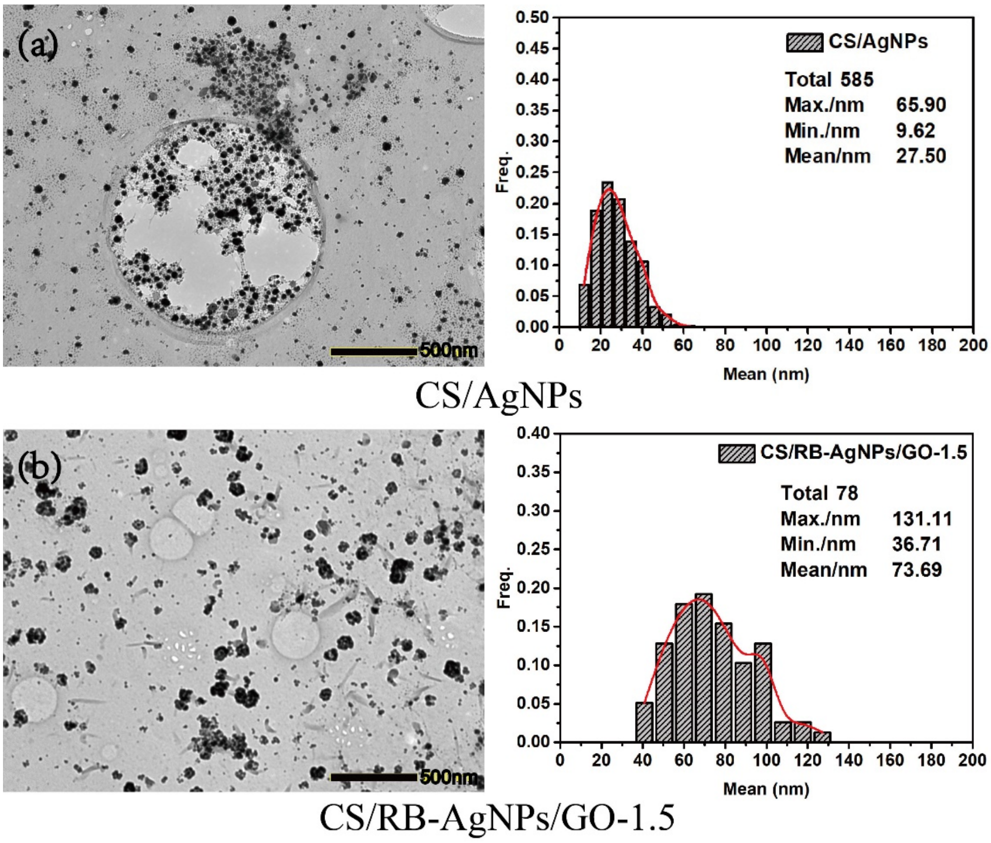


**Figure S2.** TEM images and the associated nanoparticle size distributions for CS/AgNPs and CS/RB-AgNPs/GO-1.5.

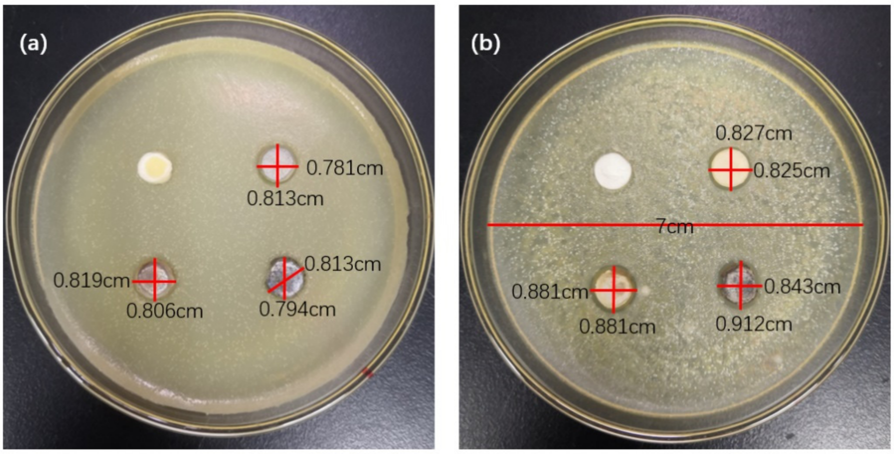


**Figure S3.** Antibacterial tests against (a) E.coli and (b) S.aureus, showing typical measurements of the diameter of the inhibition zone (inhibition length). Top left—pure CS; top right—CS/AgNPs; bottom left—CS/RB-AGNPs/GO-0.5; bottom right—CS/RB-AGNPs/GO-1.5.

**Table S2.** The kinetic parameters for different drug release models.

| Sample code | Zero order kinetic | | First order kinetic | | Higuchi model | | Korsmeyer–Peppas model | |
|---|---|---|---|---|---|---|---|---|
| | $K_0$ | $R^2$ | $K_1$ | $R^2$ | $K_H$ | $R^2$ | $N$ | $R^2$ |
| MB7.4 | 0.491 | 0.620 | -0.00216 | 0.774 | 2.631 | 0.764 | 0.121 | 0.894 |
| MB4.0 | 0.612 | 0.640 | -0.00814 | 0.730 | 3.713 | 0.769 | 0.145 | 0.910 |
| FL7.4 | 0.265 | 0.750 | -0.00087 | 0.901 | 2.116 | 0.945 | 0.179 | 0.979 |
| FL4.0 | 0.536 | 0.873 | -0.00680 | 0.952 | 6.095 | 0.977 | 0.328 | 0.971 |
| (MB/FL)-MB7.4 | 0.344 | 0.648 | -0.00098 | 0.765 | 2.044 | 0.858 | 0.127 | 0.976 |

| | | | | | | | | |
|---|---|---|---|---|---|---|---|---|
| (MB/FL)-MB4.0 | 0.459 | 0.645 | -0.00189 | 0.708 | 2.890 | 0.754 | 0.156 | 0.892 |
| (MB/FL)-FL7.4 | 0.322 | 0.690 | -0.00101 | 0.848 | 2.180 | 0.897 | 0.149 | 0.969 |
| (MB/FL)-FL4.0 | 0.587 | 0.792 | -0.00833 | 0.975 | 5.715 | 0.923 | 0.281 | 0.969 |

Note: MB7.4 = (CS/RB-AgNPs)-MB/GO-1.5 pH = 7.4; FL7.4 = CS/RB-AgNPs/(GO-1.5)-FL pH = 7.4;

MB4.0 = (CS/RB-AgNPs)-MB/GO-1.5 pH = 4.0; FL4.0 = CS/RB-AgNPs/(GO-1.5)-FL pH = 4.0;

(MB/FL)-MB7.4 = (CS/RB-AgNPs)-MB/(GO-1.5)-FL pH = 7.4 MB;

(MB/FL)-MB4.0 = (CS/RB-AgNPs)-MB/(GO-1.5)-FL pH = 4.0 MB;

(MB/FL)-FL7.4 = (CS/RB-AgNPs)-MB/(GO-1.5)-FL pH = 7.4 FL;

(MB/FL)-FL4.0 = (CS/RB-AgNPs)-MB/(GO-1.5)-FL pH = 4.0 FL.

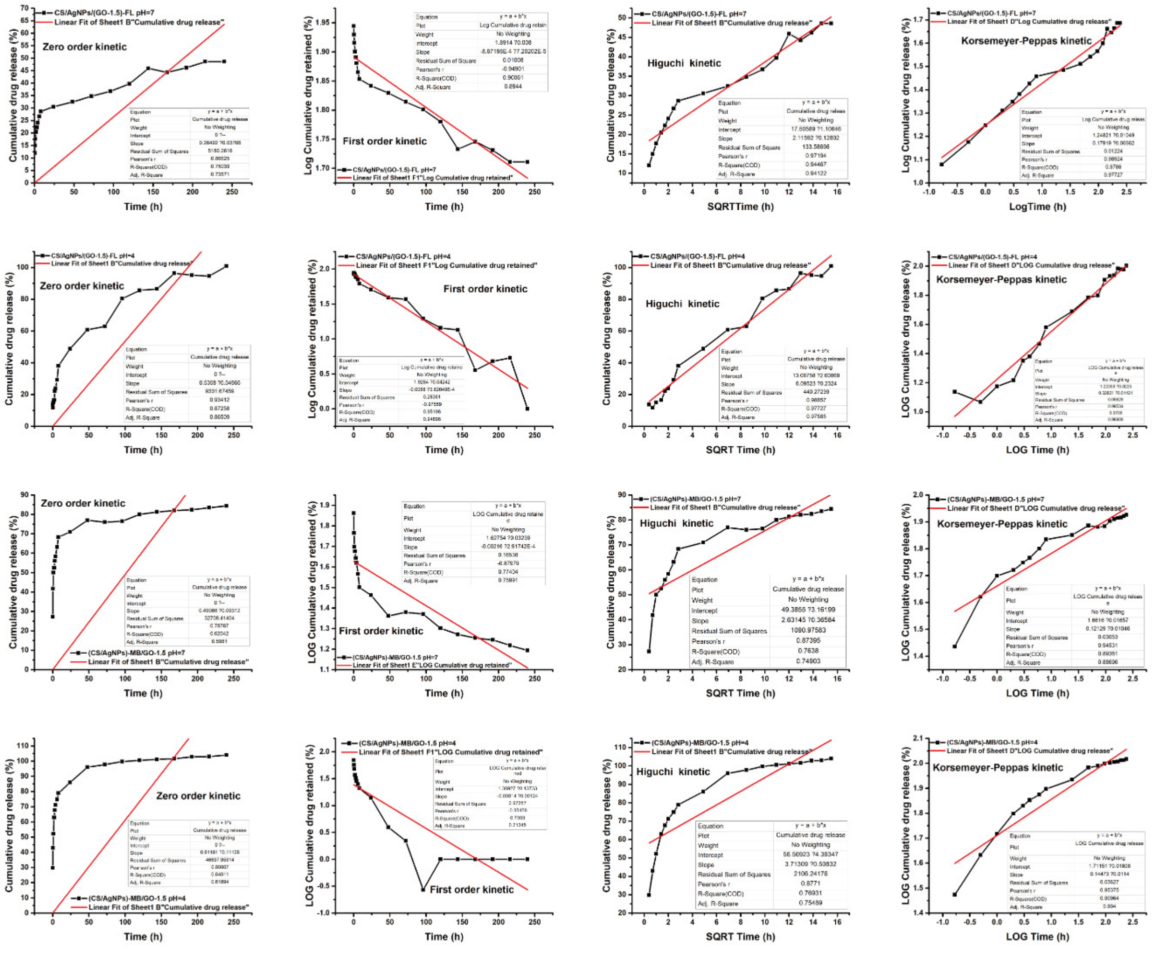


**Figure S4.** *In-vitro* release profile of single drug release system with each formulation.

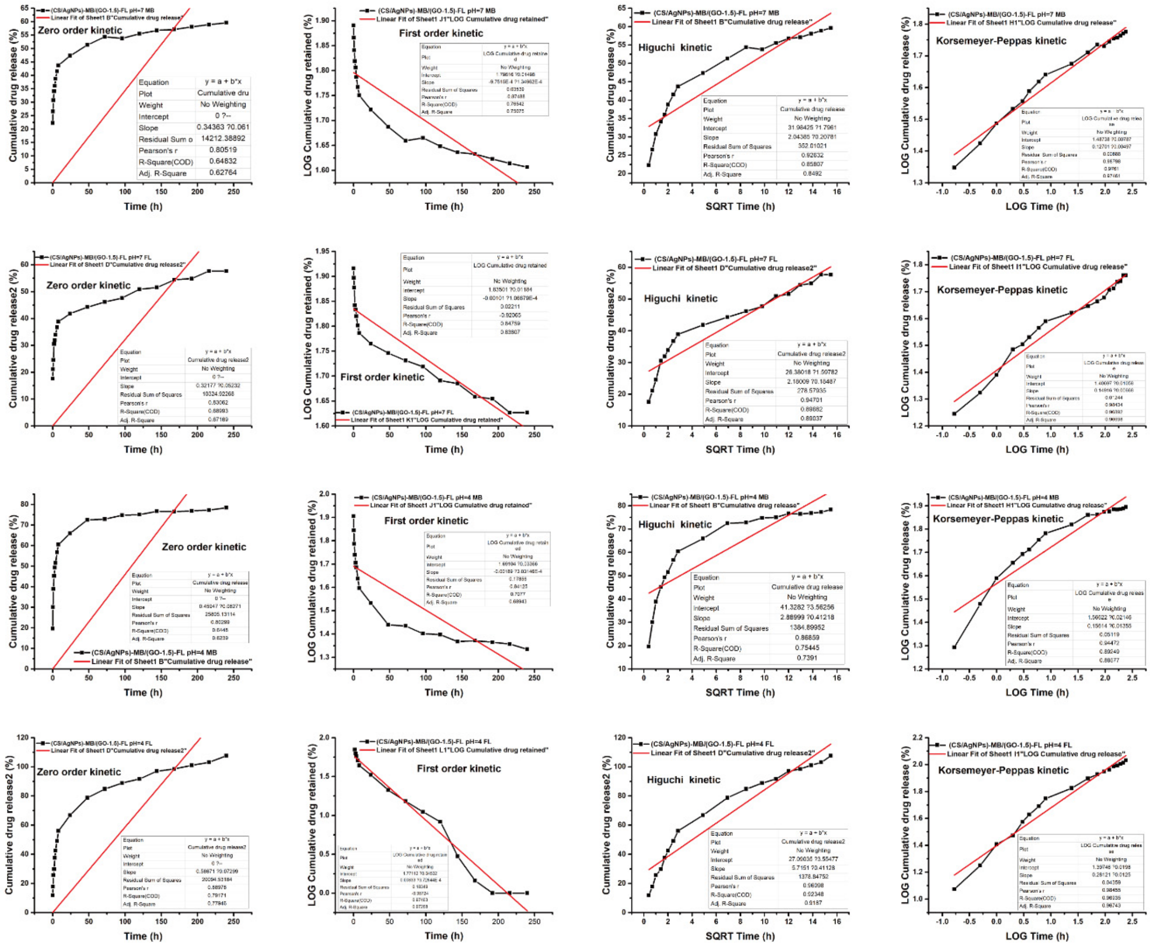


**Figure S5.** *In-vitro* release profile of dual drug release system with each formulation.

## References

1. Jia, J., Kan, C.-M., Lin, X., Shen, X., Kim, J.-K. Effects of processing and material parameters on synthesis of monolayer ultralarge graphene oxide sheets. *Carbon* **2014**, *77*, 244–254.
2. Gao, X., Tang, X. Effective reduction of graphene oxide thin films by a fluorinating agent: Diethylaminosulfur trifluoride. *Carbon* **2014**,*76*, 133–140.
3. Kou, L., Gao, C. Making silica nanoparticle-covered graphene oxide nanohybrids as general building blocks for large-area superhydrophilic coatings. *Nanoscale* **2011,** *3*, 519–528.
4. Yan, J., Wei, T., Shao, B., Ma, F., Fan, Z., Zhang, M., et al. Electrochemical properties of graphene nanosheet/carbon black composites as electrodes for supercapacitors. *Carbon* **2010**, *48*, 1731–1737.
